# Radiation awareness among radiology residents, technologists, fellows and staff: where do we stand?

**DOI:** 10.1007/s13244-014-0365-x

**Published:** 2014-11-21

**Authors:** Subramaniyan Ramanathan, John Ryan

**Affiliations:** 1 Department of Diagnostic Imaging, The Ottawa Hospital, University of Ottawa, Ottawa, Ontario Canada; 2 The Ottawa Hospital, General Campus, 501 Smyth road, Ottawa, Ontario K1S8L6 Canada

**Keywords:** Radiation dose, Radiation risk, Residents, Technologists, Cancer risk, Questionnaire

## Abstract

**Objectives:**

To investigate and compare the knowledge of radiation dose and risk incurred in common radiology examinations among radiology residents, fellows, staff radiologists and technologists.

**Methods:**

A questionnaire containing 17 multiple choice questions was administered to all residents, technologists, fellows and staff radiologists of the department of medical imaging through the hospital group mailing list.

**Results:**

A total of 92 responses was received. Mean score was 8.5 out of 17. Only 48 % of all participants scored more than 50 % correct answers. Only 23 % were aware of dose from both single-view and two-view chest X-ray; 50–70 % underestimated dose from common studies; 50–75 % underestimated the risk of fatal cancer. Awareness about radiation exposure in pregnancy is variable and particularly poor among technologists. A statistically significant comparative knowledge gap was found among technologists.

**Conclusions:**

Our results show a variable level of knowledge about radiation dose and risk among radiology residents, fellows, staff radiologists and technologists, but overall knowledge is inadequate in all groups. There is significant underestimation of dosage and cancer risk from common examinations, which could potentially lead to suboptimal risk assessment and excessive or unwarranted studies posing significant radiation hazard to the patient and radiology workers.

**Main Messages:**

• *Knowledge of radiation dose and risk is poor among all radiology workers*.

• *Significant knowledge gap among technologists compared to residents, fellows and staff radiologists*.

• *Significant underestimation of radiation dose and cancer risk from common examinations*.

## Introduction

Radiology plays a prominent role in modern medicine. Many of the diagnostic and interventional radiology procedures involve exposure to ionising radiation. Although overall the benefits of imaging outweigh the associated risks of radiation, there is growing concern over the adverse biological effects of ionising radiation on living organisms. A 2009 National Council on Radiation Protection and Measurements publication, “Ionizing Radiation Exposure of the Population of the United States”, reported a sevenfold increase in radiation exposure to the population of the United States from medical radiation since the early 1980s [1]. Stochastic effects of radiation, especially the cancer risk, is the most feared and least understood as it has no minimal threshold dosage and the adverse outcomes take at least 1–2 decades to manifest [2–4].

Review of the published scientific literature shows the knowledge of radiation dose and risk incurred in radiological examinations is very limited. Numerous studies have been performed, predominantly among physicians of different specialties, medical students and trainees, and family practitioners [5–13]. Surprisingly, there are very few studies among radiology workers. Overall these studies indicate limited knowledge in medical professionals about radiation risks incurred to patients during common imaging tests, and an inability to correctly answer the common questions raised by patients [9–12, 14–16]. It is important for the referring physicians to have adequate knowledge about radiation, as they are the ones ordering the tests in the first place. However, the radiologists have the important task of deciding on the appropriateness of the study for an individual patient and to discuss the difference in opinion and disagreements, if any, with the referring physician and the patient. It is also the duty of radiologists to answer the patient’s concerns and at the same time to impart knowledge of radiation risks to their clinical colleagues. To the best of our knowledge, no comprehensive research has been conducted exclusively among radiology workers. Hence we undertook this study to investigate and compare the level of knowledge about radiation dose and the risk incurred during common radiological examinations among radiology residents, fellows, staff radiologists and technologists.

## Materials and methods

This prospective cross-sectional study was conducted across the three campuses of a tertiary care university teaching hospital after institutional research ethics board (REB) and department medical physicist’s approval. A multiple choice questionnaire containing 17 questions on various aspects of radiation exposure was designed with an online survey tool and the link was emailed to all the residents, fellows, staff radiologists and technologists of the Department of Medical Imaging. Residents were included irrespective of their year of training. Fellows and staff radiologists were included irrespective of their subspecialties. Both computed tomography (CT) and magnetic resonance imaging (MRI) technologists were included, as their basic training is similar in radiation physics. A time limit of 2 weeks was provided to complete the questionnaire through the online survey tool and a reminder email with the link was sent 3 days before the deadline. The authors were excluded from the study. Data was collected anonymously except for the designation of the participant (resident, fellow, staff radiologist or technologist).

## Questionnaire

The questionnaire contained 17 multiple choice questions about various aspects of radiation dose and risk (“Appendix”). The first few questions assessed the basic knowledge about the average natural background radiation, units of measurement of radiation and effective dose from a chest radiograph. The effective dose an individual (adult patient) would receive from a number of common diagnostic radiological examinations in terms of chest X-ray equivalents (assuming exposure from a single-view chest X-ray as 1 arbitrary unit) was evaluated. Next, participants were asked to choose the approximate estimated risk of cancer from common radiological examinations based on four levels of risk (minimal, very low, low and moderate). One question was on management of accidental radiation exposure during pregnancy. All the questions were in multiple choice formats, with four options and only one correct answer. Levels of exposure to ionising radiation from medical imaging differ by country, institution and the imaging equipment used. The questionnaire was compiled based on the data from multiple published resources. [3, 17–22].

## Statistical analysis

Data from completed online surveys was transferred manually to Excel (Microsoft, Redmond, WA, USA) and then to SPSS, version 17.0 (SPSS, Chicago, IL, USA) for statistical analysis. Before analysis, all variables were reviewed for accuracy of data entry and missing values. Mean score was calculated out of 18 for each group (residents, fellows, staff radiologists and technologists). Overall percentage of participants who gave correct answers was computed for each question. Chi-squared test of independence was used to analyse individual questions. Mann–Whitney and Kruskall-Wallis tests were used to compare the responses among groups. The overall value for statistical significance was *P* < 0.05.

## Results

A total of 92 responses were received from 119 questionnaires sent (77 % response rate). Out of the participants, staff radiologists were the dominant group in number, followed by technologists, residents and fellows (Table 1). Overall mean score of correct answers was 8.5 out of 17 (50 %). Range of scores varied from 3 to 15 out of 17 correct answers. Mean scores of different groups are given in Table 1. Statistical difference in mean scores was found between residents and technologists, and between fellows and technologists with a *P* value of <0.05 (Table 2). The percentage of participants who scored >50 % (mean score >8.5 out of 17) was 48 % (*n* = 45).

**Table 1:** Number of participants in each group and their mean scores

Group of participants	Number of participants, *n* (%)	Mean score out of 17	Standard deviation
Residents	22 (24 %)	9.1	±2.5
Fellows	12 (13 %)	9.2	±1.6
Staff radiologists	34 (37 %)	8.5	±2.4
Technologists	24 (26 %)	7.4	±3.1
Total	92 (100 %)	8.5	±2.6

**Table 2:** Comparison of mean scores among different groups

Comparison of mean scores	*P* value
Residents vs fellows	0.53
Residents vs staff	0.154
Residents vs technologists	0.022
Fellows vs staff	0.33
Fellows vs technologists	0.034
Staff vs technologists	0.135

Out of the 17 questions, two questions were answered correctly by all the residents, fellows and staff radiologists. Twenty-one of the 24 technologists answered these two questions correctly. Those two questions are, “most sensitive age group for radiation is children” and “imaging with no radiation risk is MRI”.

Approximate effective dose from a single-view chest X-ray is 0.02 mSv and from a two-view chest X-ray is five times the single-view chest X-ray. Correct answer for single-view chest X-ray was given by 51 % (*n* = 47), for a two-view chest X-ray by 35 % (*n* = 32) and for both by only 23 % (*n* = 21) of participants. No statistical difference found among different groups of participants.

**Fig. 1:**
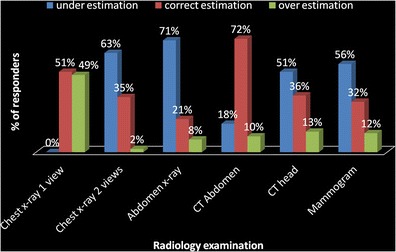
Percentage of participants who underestimated and overestimated the effective dosage equivalents of different radiology examinations

A large number of participants correctly identified the chest X-ray equivalents of CT abdomen (*n* = 66, 72 %) and single-view chest X-ray (*n* = 46, 51 %). A variable percentage of participants (20–35 %) correctly identified the chest X-ray equivalents from other common examinations. Underestimation, correct estimation and overestimation of effective dose from common examinations are shown in Fig. 1. Overall there is significant underestimation of effective dose, as demonstrated by 50–70 % of the participants underestimating the effective dosage in two-view chest X-ray, abdominal X-ray, mammogram and CT head. CT abdomen is an exception, where 72 % (*n* = 66) correctly identified the effective chest X-ray equivalents. However, still underestimation (18 %, *n* = 16) is more than overestimation (10 %, *n* = 9). Underestimation is 0 % for single-view chest X-ray, as the question did not have an option of underestimated dose.

Of the participants, 87 % (*n* = 80) correctly identified the minimal risk of cancer from chest X-ray. However, only 25–40 % of the participants correctly identified the level of cancer risk for the rest of the examinations. For whole-body PET (positron emission tomography), notably only 8 % (*n* = 7) of the participants correctly identified the moderate risk of cancer.

**Fig. 2:**
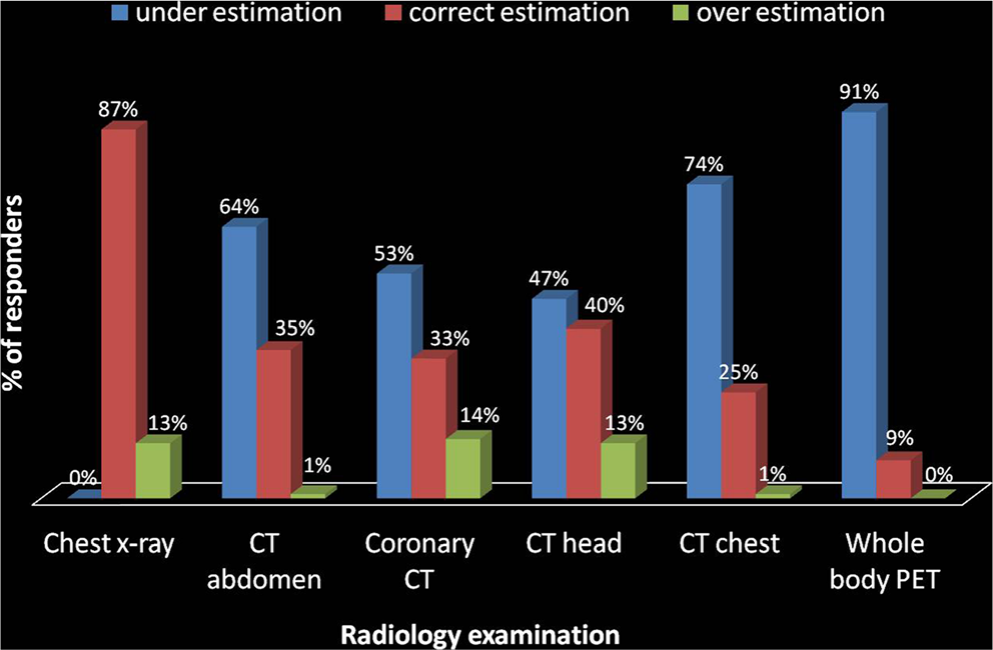
Percentage of participants who underestimated and overestimated the level of cancer risk from different radiology examinations

Correct, underestimations and overestimations of cancer risk from common examinations are shown in Fig. 2. There is significant underestimation of cancer risk as shown by 50–75 % of the participants underestimating the level of cancer risk in CT abdomen, CT head, CT chest and coronary CT. Of the participants, 91 % (*n* = 83) underestimated the cancer risk from whole-body PET. Underestimation of cancer risk is 0 % for single-view chest X-ray, as the question did not have an option of underestimated risk level.

Accidental CT abdomen performed in a pregnant woman irrespective of the trimester needs only reassurance, as the fetal risk from a single exposure is negligible [19]. This was correctly answered by 86 % (*n* = 20) of residents, 58 % (*n* = 7) of fellows and 71 % (*n* = 24) of staff radiologists. Only 13 % (*n* = 3) of the technologists selected the correct option. A statistically significant difference (*P* < 0.001) was found between technologists and residents, fellows, staff radiologists as a group and separately.

## Discussion

Our study results indicate overall poor knowledge on radiation dose and risk among radiology residents, fellows, staff radiologists and technologists of the Department of Medical Imaging. This is not different from many prior publications showing similar results among medical students, interns and physicians of various non-radiological specialties. To the best of our knowledge, no research has been performed exclusively among radiology workers. Very few studies included radiologists as a part of multiple specialities [6, 9, 11] and one recent study compared the knowledge between radiologists and non-radiologists [10].

Surprisingly, lack of large studies of radiation knowledge among radiology workers could be due to the fact that radiation dose and risks are part of their learning curriculum and the assumption that they would be experts in different aspects of radiation. It is important for the physicians to have sufficient knowledge of radiation risks, as they would be the ones requesting a radiology examination in the first place. However, the radiologists are expected to have a wider and deeper knowledge on various aspects of medical radiation exposure and should be available to guide the physicians in choosing an appropriate imaging modality that would provide the optimal answer to the clinical question with minimal radiation hazard.

Our study results reflect a serious knowledge deficit among each of the different groups of radiology workers. Though the knowledge of radiologists reported in our study is slightly better than quoted for physicians of other specialities in prior studies, it is still inadequate [5, 6, 8–10]. Nearly half of our study group scored less than 50 % on questions of radiation dose and risks. Although the study group is not homogeneous, we found statistically significant differences in the mean score among technologists compared with residents and fellows. One of the most frequently performed radiology examination is chest X-ray and nearly half of the participants did not know about the dosage from a single-view chest X-ray. This is not different from prior publications [5, 6, 10, 12]. Unfortunately only one-fourth of the participants had knowledge about the radiation dosage from both single-view and two-view chest X-rays and their relationship, indicating an important knowledge gap. This is comparable to a recent study which revealed that only 32 % of radiologists identified the correct dosage of chest X-ray [10].

On the whole, better knowledge of radiation exposure from CT abdomen was found among all the groups indicated by 72 % of the participants correctly identifying the chest X-ray equivalents of CT abdomen. This is in contrast to a small prospective study in 2004, where only 13 % of radiologists identified the same [9]. This could be due to various reasons, like the rapid increase in usage of multi-detector CT in the last 10 years, CT abdomen now being one of the commonly performed studies with significant exposure and probably increased awareness of radiation risk. Except for CT abdomen, there is, however, a significant underestimation of dosage from other common examinations. There is also significant underestimation of cancer risk as expected from significant underestimation of dosages. Interestingly, for whole-body PET, only 8 % correctly identified the level of cancer risk and nearly 90 % underestimated the risk. This might be explained by the fact that our group consisted of only radiologists who do not practice nuclear medicine, and nuclear medicine is a separate department. This knowledge deficit of underestimating cancer risk of commonly performed examinations is of serious concern, as it may lead to acceptance of many unwarranted examinations from physicians and repeat studies, which all add up to significant radiation hazard and major public health concern.

One hundred percent of residents, fellows, staff radiologists and >90 % of technologists correctly identified the absence of radiation risk in MRI and increased radiation risk in the paediatric population. This is in strong contrast to prior studies among physicians reporting variably poor knowledge. This is important, as the radiologist with adequate knowledge about the exposures in different modalities can suggest appropriate alternate imaging options, depending on the clinical question and patient’s age.

Lastly, the subject of radiation exposure in pregnancy is complex and risk benefit ratio needs to be considered carefully before proceeding with the examination. Radiologists play a prominent role in deciding the appropriate imaging modality based on the trimester, clinical question and availability. In our study, though very limited, knowledge of radiation risk in pregnancy was assessed based on a single question (Appendix, question no. 17). Importantly, only 13 % of technologists gave the correct answer, and a significant proportion of the participants suggested medical termination of pregnancy as an option. The knowledge was variable among other groups (residents, fellows and staff radiologists) in the range of 60–85 %. This is highly important, as the technologists come into close contact with the patients in the radiology department and they should have adequate knowledge on radiation exposure during pregnancy and should ideally be trained enough for answering patients’ concerns and arranging a discussion with the radiologist.

Our study suffered from a number of limitations. It is a single tertiary care institutional study and this may limit extrapolation of the results to different settings, especially small community and non-teaching hospitals. Our sample size, although not very small, it is not large enough and needs countrywide studies before taking major actions. Our questionnaire is limited to 17 questions focusing mainly on radiation dose and cancer risk of common examinations. Ideally it cannot be equated to comprehensive radiation knowledge. Many of the questions asked about precise numerical answers which were felt not practicable by many of our participants. However, the authors were of the opinion that we, as radiology workers, are expected to have deeper and more accurate knowledge on radiation dose and cancer risks and this needs to be imparted in the early stages of radiology training. Few of the questions were interrelated and theoretically it was possible to deduce the answers from other questions. Few questions on effective dose and cancer risk have a wide range of variable answers depending on the source of information. As the survey was performed online with a 2-week time limit, there exists potential opportunity to research for correct answers from various resources. All these limitations could potentially skew the real knowledge status. However, with results showing significant knowledge gaps, the real or true knowledge of radiation could be even worse than evaluated. As the data were collected anonymously, the difference in the knowledge among residents in different years of training, fellows and staff radiologists of different subspecialties could not be evaluated.

It is important that we take knowledge about radiation dose and risk more seriously. Many of our subspecialty leads and Chair of the Medical Imaging Department were surprised and disappointed with the results. We do believe that our institution is not alone in this battle and unfortunately currently there are no published data on radiation knowledge to compare with other teaching institutions in North America. We are trying to enforce many of the recommendations of the American College of Radiology blue ribbon panel, which includes improving medical physics training during residency, including radiation safety topics in exit examinations, regular in-service training for technologists on radiation safety, which we are currently conducting every 3 months, and advanced training of selected enthusiastic technologists who can impart periodic training to other staff [17]. Periodic continuous medical educational (CME) activities are recommended among radiology workers [23] and we are working to make this mandatory for all, including the staff radiologists irrespective of subspecialties, to update themselves on radiation dosage and risks and provide the evidence of acquired CME credits. This could help in providing optimal usage of imaging resources and minimising the unpredictable and apparently unavoidable risk of cancer, albeit very small. Pre- and post-educational session assessment can be performed to assess improvement by these endeavours. Other measures such as including the patient’s total radiation exposure in the imaging report, and including the radiation dosages in the radiology request forms could also create greater awareness among physicians and patients, and potentially reduce the injudicious usage of imaging, although this needs extensive discussion among physicians and patients for ethical concerns and practical difficulties.

## Conclusions

Radiologists are expected to have pertinent knowledge to guide the referring physicians in selecting the appropriate imaging modality, based on their training in radiation dose, risk and safety. Disappointingly, the results of this survey show significant knowledge deficit among all radiology workers, including residents, fellows, staff radiologists and technologists. Overall there is significant underestimation of dosage and cancer risk from common examinations. Inaccuracy is seen even in estimating the dosage of commonly performed chest X-rays. Although the questionnaire was not an all-inclusive one and not an ideal way of knowledge assessment with numerous limitations as detailed above in the “Discussion”, significant selective knowledge deficit was identified on typical dose levels and estimated risks of cancer induction of several important imaging examinations. Statistically significant knowledge deficit was seen in technologists compared with residents, fellows and staff radiologists as a group. This is of concern as technologists are the first point of contact with the patients and they should be adequately trained to answer common patient questions and concerns. The next level of contact is the residents and fellows, who are often called upon to advise colleagues in other specialities and patients about dose and safety concerns. Staff radiologists have the most important role of acquiring and imparting the knowledge about radiation and any updates in the field to the technologists, residents and fellows periodically, and to provide expert counsel on risk and dose issues. Based on our results, a conscientious effort to provide more robust education and acquire greater knowledge in these matters is required.

## Appendix

**Questionnaire on radiation risk and doses** (Correct answers are highlighted in *italics*).

1. What is your current designation in the radiology department?
  - Resident
  - Fellow
  - Staff radiologist
  - Technologist
2. Average natural background radiation is in the range
  - 20–30 mSv
  - *2*–*3 mSv*
  - 0.2-0.3 mSv
  - 200–300 mSv
3. Approximate effective dose received by a patient in a single-view chest X-ray is
  - 0.5 mSv
  - 1 mSv
  - *0.02 mSv*
  - 0.05 mSv
4. Approximate effective dose received by a patient in a two-view chest X-ray is
  - Almost equal to single-view chest X-ray
  - Twice the single-view chest X-ray
  - *5 times the single-view chest X-ray*
  - 10 times the single-view chest X-ray
5. Effective dose from a single-view AXR is equivalent to
  - 0–1 cxr
  - 1–10 cxr
  - *10*–*50 cxr*
  - 50–100
6. CT abdomen single phase gives a dose of
  - *10 mSv*
  - 100mSv
  - 1 mSv
  - None
7. Dose from a CT abdomen is equivalent to dosage from
  - 10–100 chest X-rays
  - *100*–*500 chest X-rays*
  - More than 1,000 chest X-rays
  - 1 chest X-ray
8. Dose from a CT Head is equivalent to dosage from
  - 10–50 chest X-rays
  - *50*–*100 chest X-rays*
  - 100–500 chest X-rays
  - 10 chest X-rays
9. Dosage from two-view unilateral mammogram is
  - Almost equal to single-view chest X-ray
  - Twice the single-view chest X-ray
  - *10*–*20 times the single-view chest X-ray*
  - 50–100 times the single-view chest X-ray
10. Which of the following has no radiation risks:
  - Fluoroscopy
  - *MRI*
  - PET
  - Technetium bone scan
11. Please select which one of the following is most sensitive to radiation:
  - *Children*
  - Adolescents
  - Adults
  - Elderly
12. Approximate estimated risks of fatal cancer from CXR
  - *Minimal: 1 in 1,000,000 to 1 in 100,000*
  - Very low: 1 in 100,000 to 1 in 10,000
  - Low: 1 in 10,000 to 1 in 1,000
  - Moderate: 1 in 1,000 to 1 in 500
13. Approximate estimated risks of fatal cancer from CT abdomen
  - Minimal: 1 in 1,000,000 to 1 in 100,000
  - Very low: 1 in 100,000 to 1 in 10,000
  - *Low: 1 in 10,000 to 1 in 1,000*
  - Moderate: 1 in 1,000 to 1 in 500
14. Approximate estimated risks of fatal cancer from Coronary CT angiography
  - Minimal: 1 in 1,000,000 to 1 in 100,000
  - Very low: 1 in 100,000 to 1 in 10,000
  - *Low: 1 in 10,000 to 1 in 1,000*
  - Moderate: 1 in 1,000 to 1 in 500
15. Approximate estimated risks of fatal cancer from CT head
  - Minimal: 1 in 1,000,000 to 1 in 100,000
  - *Very low: 1 in 100,000 to 1 in 10,000*
  - Low: 1 in 10,000 to 1 in 1,000
  - Moderate: 1 in 1,000 to 1 in 500
16. Approximate estimated risks of fatal cancer from Whole-body PET
  - Minimal: 1 in 1,000,000 to 1 in 100,000
  - Very low: 1 in 100,000 to 1 in 10,000
  - Low: 1 in 10,000 to 1 in 1,000
  - *Moderate: 1 in 1000 to 1 in 500*
17. Approximate estimated risks of fatal cancer from CT chest:
  - Minimal: 1 in 1,000,000 to 1 in 100,000
  - Very low: 1 in 100,000 to 1 in 10,000
  - *Low: 1 in 10,000 to 1 in 1,000*
  - Moderate: 1 in 1,000 to 1 in 500
18. A pregnant woman underwent CT abdomen and pelvis with contrast as her pregnancy status was not enquired by the CT technologist before performing CT. What should be the course of action according to ACR guidelines?
  - *Reassure the mother that the risk to the fetus is negligible*
  - Suggest medical termination of pregnancy as an option
  - Do genetic analysis by amniocentesis or chorionic villous biopsy
  - Do MRI of the fetus to look for CNS anomalies.
